# Complete Genome Sequences of Five *Paenibacillus larvae* Bacteriophages

**DOI:** 10.1128/genomeA.00668-13

**Published:** 2013-11-14

**Authors:** Michael A. Sheflo, Adam V. Gardner, Bryan D. Merrill, Joshua N. B. Fisher, Bryce L. Lunt, Donald P. Breakwell, Julianne H. Grose, Sandra H. Burnett

**Affiliations:** Department of Microbiology and Molecular Biology, Brigham Young University, Provo, Utah, USA

## Abstract

*Paenibacillus larvae* is a pathogen of honeybees that causes American foulbrood (AFB). We isolated bacteriophages from soil containing bee debris collected near beehives in Utah. We announce five high-quality complete genome sequences, which represent the first completed genome sequences submitted to GenBank for any *P. larvae* bacteriophage.

## GENOME ANNOUNCEMENT

*Paenibacillus larvae* is a facultative anaerobic spore-forming pathogen that causes American foulbrood (AFB). AFB kills honeybee larvae (1), contributes to colony collapse disorder (2), and limits agricultural yields (3). Unfortunately, some *P. larvae* strains have become resistant to the antibiotics typically used for AFB treatment (4). Phage therapy is a potential treatment for AFB, yet few *P. larvae*-specific phages have been described (5–7), and a full-genome sequence for one has only recently become available (8).

Soil, honey, and larva samples were collected in the Utah, Salt Lake, and Davis counties of Utah. The samples were used for isolating new host strains or bacteriophages. *P. larvae* subsp. *pulvifaciens* (9) hosts were confirmed by 16S rRNA sequencing. The bacterial cultures were inoculated with soil samples to enrich for bacteriophages. Plated enrichment samples formed plaques from which bacteriophages were selected and purified by a minimum of three passages. High-titer lysates were filtered, incubated with 5 µg/ml RNase and 10 µg/ml DNase for 30 min at 37°C, and treated with 100 µg/µl proteinase K at 52°C for 1 h. Following phenol-chloroform extraction and ethanol precipitation, high-quality DNA (10) was sequenced using 454 pyrosequencing.

Raw sequences were assembled into contigs using Newbler version 2.6 (Roche Diagnostics, Branford, CT) and Consed version 19 (11). The phages Abouo and Emery assembled into single contigs. The phages Jimmer1, Jimmer2, and Davies assembled into multiple contigs that were joined using Gepard 1.30 (12), MEGA5 (13), and Geneious Pro 5.4.4 (Biomatters Ltd., Auckland, New Zealand) with phage Abouo as a reference. The sequencing fold-coverage data are provided in Table 1.

**TABLE 1:** *Paenbacillus larvae* bacteriophage genomes

Phage name	GenBank accession no.	Sequencing fold coverage	Length (bp)	No. of genes	G+C content (%)
Jimmer1	KC595515	250.2	54,312	100	38.11
Jimmer2	KC595514	271.5	54,312	100	38.10
Emery	KC595516	143.2	58,572	100	41.44
Abouo	KC595517	116.9	45,552	92	39.16
Davies	KC595518	130.8	45,798	93	39.16

No experiments were performed to determine the physical ends or the packing or replication strategies of the phage DNA. However, during manual finishing, overlapping contigs assembled the genome into an apparently circular genome. For the bacteriophages Jimmer1, Jimmer2, Davies, and Abouo, the first base of each genome was selected in the noncoding gap between the terminase gene and the prior gene. Since Emery did not have a terminase small subunit gene, the first base was selected in the first gap upstream of the large subunit terminase gene. The noncoding gap where the first base was selected for a genome contained multiple stops and lacked coding potential in any frame in all genomes.

Annotation was completed using DNA Master (http://cobamide2.bio.pitt.edu). A coding potential map was generated using GeneMark 2.5p (14) for each phage based on *Bacillus cereus* strain ATCC 14579, the closest available relative to *P. larvae*. Our selection of gene calls emphasized the following criteria: GeneMark HHM and Glimmer autoannotation, BLAST alignment *E* values of <0.001, coding potential from GeneMark, start codon sequences, and Shine-Dalgarno (SD) scores of >200 nats using the Karlin position-specific scoring matrix (PSSM) for moderately to highly expressed genes.

All five phages were identified as myoviruses. Jimmer1 and Jimmer2 were isolated independently from the same soil sample and differ in their genomic sequences by only 80 bp of the 54,312-bp genomes (99.85% similarity). The differences between these related phages are real base pair changes because the sequencing fold coverages of these samples are statistically greater than the error rate of 454 sequencing (15).

### Nucleotide sequence accession numbers.

The GenBank accession numbers for the five *Paenibacillus larvae* bacteriophages are listed in Table 1.
