# Correction for Sheflo et al., Complete Genome Sequences of Five *Brevibacillus laterosporus* Bacteriophages

**DOI:** 10.1128/genomeA.01113-15

**Published:** 2015-10-01

**Authors:** Michael A. Sheflo, Adam V. Gardner, Bryan D. Merrill, Joshua N. B. Fisher, Bryce L. Lunt, Donald P. Breakwell, Julianne H. Grose, Sandra H. Burnett

**Affiliations:** Department of Microbiology and Molecular Biology, Brigham Young University, Provo, Utah, USA

## AUTHOR CORRECTION

Volume 1, no. 6, e00668-13, 2013. The species infected by the five bacteriophages was misidentified as *Paenibacillus larvae*. The article title should read as given above, and the organism identified as *Paenibacillus larvae* should be identified as *Brevibacillus laterosporus* throughout.

Page 1: The abstract should read as follows. “*Brevibacillus laterosporus* is a secondary invader following European foulbrood (EFB) infection of honeybees. We isolated bacteriophages from soil containing bee debris collected near beehives in Utah. We announce five high-quality complete genome sequences, which represent the first completed genome sequences submitted to GenBank for any *B. laterosporus* bacteriophage.”

Page 1: The first paragraph of the text should read as follows. “*Brevibacillus laterosporus* is an anaerobic, spore-forming bacterium that invades beehives following European foulbrood (EFB) infection (1). Honeybee bacterial diseases, including EFB and American foulbrood (AFB), kill honeybee larvae, contribute to colony collapse disorder (2), and limit agricultural yields (3). Phage therapy is a potential treatment for bee foulbroods, yet few *P. larvae*-specific phages have been described (5-8), and prior to this work there was no research investigating *B. laterosporus* phages.”

Page 1, column 1: Lines 12 and 13 should read as follows. “The 16S rRNA sequencing showed similarity to two *Paenibacillus larvae* subsp. *pulvifaciens* (9) isolates, but further tests indicated that these samples were *B. laterosporus*.” 

Page 1, column 2: Lines 16-18 should read as follows. “A coding potential map was generated using GeneMark 2.5p (14) for each phage based on *Bacillus cereus* ATCC 14579, the closest available relative to *B. laterosporus*.”

Page 2: Reference 1 should be replaced with the following.

1. **Forsgren E.** 2010. European foulbrood in honey bees. J Invertebr Pathol **103**(Suppl 1)**:**S5–S9.

Page 2: Reference 4 should be deleted.

